# An augmented wood-penetrating structure: Cicada ovipositors enhanced with metals and other inorganic elements

**DOI:** 10.1038/s41598-019-56203-6

**Published:** 2019-12-24

**Authors:** Matthew S. Lehnert, Kristen E. Reiter, Gregory A. Smith, Gene Kritsky

**Affiliations:** 1 Department of Biological Sciences, Kent State University at Stark, North Canton, OH 44720 USA; 2 Department of Entomology, University of Illinois at Urbana-Champaign, Urbana, IL 61801 USA; 3 School of Behavioral and Natural Sciences, Mount St. Joseph University, Cincinnati, OH 45233 USA

**Keywords:** Ecology, Evolution

## Abstract

Few insect species are as popular as periodical cicadas (*Magicicada* spp.). Despite representing an enormous biomass and numbers that exceed 370/m^2^ during mass emergences, the extended time period of the underground nymphal stages (up to 17 years) complicates investigations of their life history traits and ecology. Upon emergence, female cicadas mate and then use their ovipositors to cut through wood to lay their eggs. Given the ability to penetrate into wood, we hypothesized that the ovipositor cuticle is augmented with inorganic elements, which could increase hardness and reduce ovipositor fracturing. We used scanning electron microscopy and energy dispersive x-ray spectroscopy to evaluate the material properties of ovipositors of four cicada species, including three species of periodical cicadas. We found 14 inorganic elements of the cuticle, of which P, Ca, Si, Mg, Na, Fe, Zn, Mn, Cl, K, and S show the highest concentrations (%wt) near the apex of the ovipositor, where other structural modifications for penetrating wood are present. To the best of our knowledge, this is the first report of metal deposits in the cuticle of true bugs (Hemiptera, >80,000 described species).

## Introduction

The independent origin of traits that perform similar functions represents a cornerstone of natural selection. Examples of such convergent evolution can be found across animal taxa: intelligence among birds and apes^1^, echolocation among bats and dolphins^2^, and fluid-feeding mechanisms among flies and butterflies^3^. A compelling example of convergence among some arthropods and annelids is the deposition of metals and other inorganic elements within cuticular structures prone to wear or abrasion^4–6^.

Natural selection has favored metal-reinforced cuticle on an array of structures that might be susceptible to wear, including insect mandibles^7–9^, insect ovipositors^6,10^, spider fangs^11^, and jaws of marine polychaetes^12–15^. Within the class Insecta, metals have been found in the cuticle of some Blattodea, Orthoptera, Phasmatodea, Lepidoptera, Hymenoptera, Diptera, and Coleoptera^6–8,15–18^. The metals found in insect cuticle are diverse, including manganese, zinc, iron, calcium, among others^10,15,16,18,19^, which are sometimes coupled with halogens, such as chlorine^4,12^.

The presence of metals in the cuticle is of particular interest because the functionality of the metal-augmented structures is closely tied to life history traits and fitness. Metals in the ovipositors of some species of wasps (Hymenoptera), for example, enhance the material properties of the cuticle, thus facilitating the ability to penetrate through wood for subsequent oviposition on select hosts^10^. The process of drilling through wood for oviposition is complex and requires a suite of morphological, behavioral, and cuticular adaptations^6,20^. Cicadas (Hemiptera), similarly, oviposit in wood; therefore, natural selection might favor augmented material properties of the ovipositor cuticle, possibly through inorganic element enhancement. Here, to the best of our knowledge, we present the first study of the inorganic elemental composition and metal deposits along the ovipositors of annual and periodical cicadas.

Periodical cicadas are the bugs of history. Observations of cicada ovipositors began in early colonial America (see Supplementary Note for historical accounts of cicada ovipositors)^21^, but the first detailed description and illustrations of the ovipositor were published in an 1839 pamphlet by Nathanial Potter^22^ (Supplementary Fig. S1). Marlatt^23^ and Hyatt^24^ described how the parts of the ovipositor’s shaft work together to cut into the twig and to lay the eggs, and Snodgrass^25^ in his classic book, *Principles of Insect Morphology*, provided terminology for these parts. The ovipositor shaft (as Potter noted) consists of three parts, two outer first valvulae (1V*l*), as named by Snodgrass, and a central rod, which Snodgrass noted consisted of the fused second valvulae (2V*l*) (Supplementary Fig. S1). The two 1V*l* possess the serrated cutting saw at the outside tip with the inner surface acting as a rasp, and also deposit the eggs into the eggnest. Subsequent studies of cicada ovipositors updated the terminology, referring to the 1V*l* and 2V*l* as the gonapophyses VIII (GVIII) and gonapophyses IX (GIX), respectively^26,27^.

Cicada oviposition has been studied with respect to their morphology, ecology, and life history traits^27–30^; however, there are few studies that discuss the mechanism of the oviposition process at the proximate level, and no studies of the material properties of cicada ovipositors. The overall aim of this study is to determine if cicada ovipositors have inorganic components in their cuticle, which could facilitate the penetration of wood for oviposition, and if so, where these elements are located.

## Results

### Morphology of cicada ovipositors

We selected four species of cicada (Hemiptera: Cicadidae), Linne’s annual cicada, *Neotibicen linnei* (Cicadinae), and three species of 17-year periodical cicada, *Magicicada septendecim* (Cicadettinae), *M. cassinii*, and *M. septendecula* to study ovipositor architecture and material properties. Measurements from images acquired with a digital microscope revealed that ovipositor lengths differed among species (*F* = 19.848; df = 3, 23; p < 0.0001). Analysis of covariance indicated that forewing length was a significant covariate of ovipositor length (*F* = 9.56; df = 3, 26; p < 0.0001), which suggests that ovipositor lengths differ, even when controlling for body size (Supplementary Table S1).

**Figure 1:**
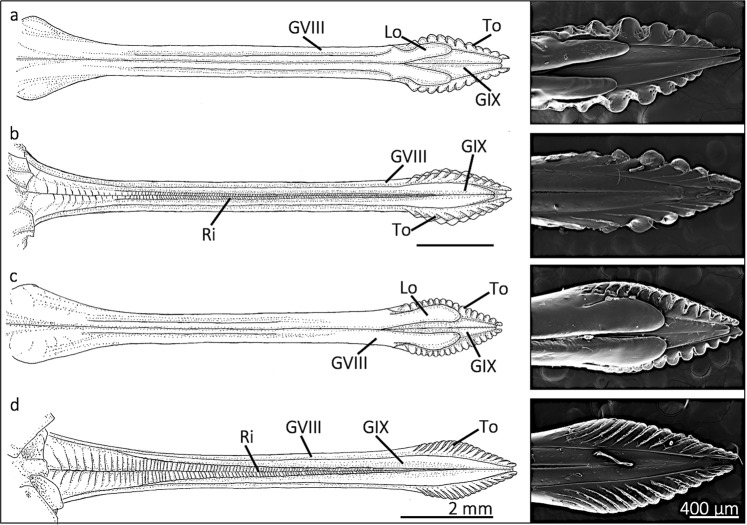
Morphology of cicada ovipositors and the rasping regions. (**a**–**d**) are illustrations of cicada ovipositors and their corresponding rasping regions are shown as SEM images on the right side. (**a**,**b**) show the ventral side and dorsal side of the ovipositor of *N. linnei* and (**c**,**d**) show the ventral and dorsal side of *M. cassinii*, respectively. All studied ovipositors consist of medial gonapophyses IX (GIX) and lateral gonapophyses VIII (GVIII). The GVIII covers the lateral sides of the GIX and wraps around the ventral side, covering the entire GIX except at the distal end. The ventral side of the GVIII has lobes (Lo) near the distal tip, which represents where the eggs exit the ovipositor. The medial region of a GIX has ridges (Ri) that interlock with ridges from the other GIX. All studied ovipositors have a rasping region with teeth (singular = tooth, To). The ovipositors were illustrated by Brooke Pandrea.

Scanning electron microscopy (SEM) revealed that all studied ovipositors consisted of two, scabbard-like GVIII that cradle the two, interconnected GIX (Fig. 1). We term the distal tip of the GVIII the “rasping region”, which is characterized by teeth (singular = tooth). The teeth are interspersed by grooves that extend proximally and medially (Fig. 1). The total width of the ovipositor differed among species along the ovipositor length (middle of ovipositor, *F* = 3.157; df = 3, 23; p = 0.044; base of rasping region, *F* = 33.53; df = 3, 23; p < 0.0001; middle of rasping region, *F* = 6.99; df = 3, 23; p = 0.002; distal part of rasping region, *F* = 4.313; df = 3, 23; p = 0.015) (locations of measurements shown in Supplementary Fig. S2). The width measurements along the rasping region length indicated differences in their overall shape among species (Fig. 1), and *N. linnei* was significantly narrower (Supplementary Table S1).

**Figure 2:**
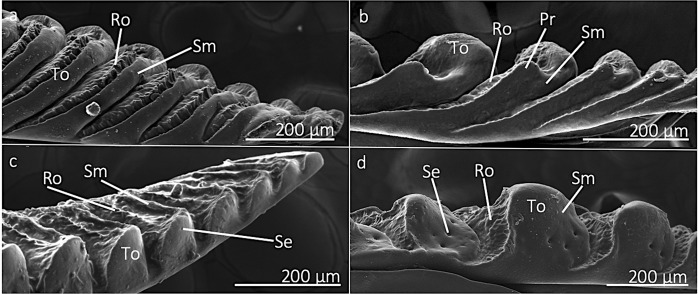
SEM images of the teeth of cicada ovipositors. (**a**,**b**) show the dorsal side of the rasping region of the ovipositor of *M. septendecula* and *N. linnei*, respectively. A tooth (To) consists of two structurally-defined regions, a smooth (Sm) distal part and a rough (Ro) proximal region. The dorsum of the rasping teeth of *N. linnei* (**b**) have a knob-like protrusion (Pr). Ventral images of ovipositors of *M. septendecim* (**c**) and *N. linnei* (**d**) show that the teeth are bump-like laterally and have campaniform sensilla (Se), which were observed in all studied species.

The length of the rasping region also differed among species (F = 33.88; df = 3, 23; p < 0.0001) and was longest on *N. linnei*; however, this species had the fewest number of teeth (F = 131.71; df = 3, 23; p < 0.0001) (SEM images in Fig. 1). A tooth has a general convex shape and consists of two distinct regions based on surface roughness patterns, a rough proximal region and a smooth distal region (Fig. 2). The rough region has a central, ridge-like area, which was particularly noticeable on the *Magicicada* spp. ovipositors. The lateral side of a tooth is knob-like, ending in an enlarged bump with campaniform sensilla (Fig. 2). The *Magicicada* spp. have rasping teeth that appear similar, whereas those of *N. linnei* do not have as prominent of an abrasive region, but have a large, and distally smooth protrusion on the dorsal side of each tooth (Fig. 2).

**Figure 3:**
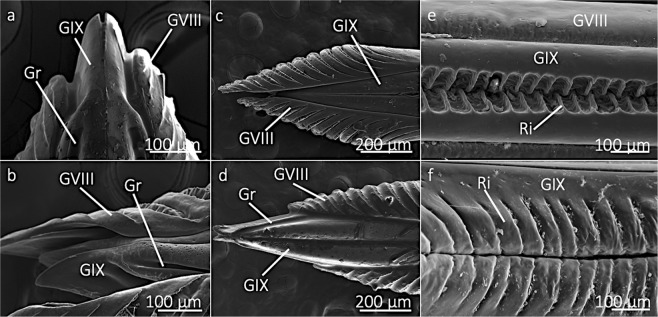
Ovipositor structures associated with the sliding mechanism. (**a**,**b**) show that the gonapophyses IX (GIX) have grooves (Gr) that fit onto the gonapophyses VIII (GVIII), allowing the GVIII to make anti-parallel movements along the GIX. The GVIII are capable of sliding distally, past the GIX (**c**), or sliding proximally, exposing the distal part of the GIX (**d**). The GIX have ridges (Ri) that are tooth-like in the distal regions (**e**) and more plate-like in proximal regions (**f**), which likely stabilize the GIX while the GVIII are performing anti-parallel movements to penetrate wood for subsequent oviposition.

The GIX has a lateral groove, which fits into grooves and ridges of the GVIII (Fig. 3). The GIX also have medial ridges, which were observed for approximately 90% of the proximal ovipositor length (Figs. 1 and 3). The shapes and sizes of the medial ridges changes along the ovipositor length and are plate-like near the proximal region, but are tooth-like and interlocking in the distal region (Figs. 1 and 3).

### The presence of inorganic elements in the cuticle of cicada ovipositors

We analyzed ten dorsal and seven ventral locations on the GVIII and GIX along the ovipositor length using energy dispersive x-ray spectroscopy (EDS) (see Supplementary Fig. S2 for locations) and detected fourteen inorganic elements: aluminum (Al), calcium (Ca), chlorine (Cl), copper (Cu), iron (Fe), potassium (K), magnesium (Mg), manganese (Mn), sodium (Na), phosphorus (P), sulfur (S), silicon (Si), zinc (Zn), and zirconium (Zr). All elements were detectable, in at least trace amounts, in all four species, except for Cu, which was not detected in *M. septendecula*. K, S, Cl, and Mn, were abundant elements across all species, while Na, Fe, Zn, and Cu were the least abundant (Table 1). Body size was not correlated with the concentration of all detected inorganic elements. Further, a correlation of PCA Axis 1 of body size data (based on forewing length measurements) with PCA Axis 1 of EDS data was not significant (Pearson coeff. = 0.446, p = 0.146), indicating that body size was not a predictor of elemental composition. A plot of individual cicadas as a function of element concentration in ordination space showed no specific groupings by species (Supplementary Fig. S3); therefore, elemental composition is not a predictor of species identity.

**Table 1:** Mean inorganic element composition (%wt) within the cuticle of the ovipositors of four cicada species.

*Element*	*M. cassinii*	*M. septendecim*	*M. septendecula*	*N. linnei*
Al	0.08 ± 0.02 (0.00, 1.99)	**0.22** ± **0.08 (0.00, 7.86)**	0.18 ± 0.06 (0.00, 3.47)	0.12 ± 0.04 (0.00, 3.68)
Ca	0.14 ± 0.02 (0.00, 1.32)	0.05 ± 0.01 (0.00, 0.80)	0.03 ± 0.01 (0.00, 0.31)	0.15 ± 0.02 (0.00, 1.70)
Cl	**0.27** ± **0.03 (0.00, 2.03)**	0.16 ± 0.02 (0.00, 1.38)	**0.53** ± **0.08 (0.00, 4.34)**	**0.32** ± **0.04 (0.00, 4.94)**
Cu	0.00 ± 0.00 (0.00, 0.01)	0.00 ± 0.00 (0.00, 0.19)	0.00 ± 0.00 (0.00, 0.00)	0.00 ± 0.00 (0.00, 0.17)
Fe	0.02 ± 0.01 (0.00, 0.37)	0.01 ± 0.03 (0.00, 0.30)	0.03 ± 0.02 (0.00, 1.54)	0.02 ± 0.01 (0.00, 0.62)
K	**0.49** ± **0.05 (0.03, 2.98)**	**0.93** ± **0.24 (0.04, 22.79)**	**1.04** ± **0.20 (0.06, 9.17)**	**1.19** ± **0.16 (0.07, 10.78)**
Mg	0.04 ± 0.01 (0.00, 0.69)	0.06 ± 0.01 (0.00, 0.31)	0.07 ± 0.01 (0.00, 0.71)	0.06 ± 0.01 (0.00, 0.48)
Mn	**0.31** ± **0.06 (0.00, 4.05)**	0.02 ± 0.00 (0.00, 0.45)	0.00 ± 0.00 (0.00, 0.00)	**0.45** ± **0.09 (0.00, 9.51)**
Na	0.01 ± 0.00 (0.00, 0.09)	0.01 ± 0.00 (0.00, 0.11)	0.08 ± 0.00 (0.00, 0.08)	0.05 ± 0.01 (0.00, 0.48)
P	0.11 ± 0.02 (0.00, 2.06)	**0.19** ± **0.02 (0.00, 1.79)**	**0.20** ± **0.04 (0.00, 1.99)**	0.03 ± 0.01 (0.00, 0.84)
S	**0.29** ± **0.03 (0.00, 2.21)**	**0.32** ± **0.05 (0.00, 5.41)**	**0.53** ± **0.06 (0.00, 2.93)**	**0.28** ± **0.02 (0.00, 1.57)**
Si	0.05 ± 0.16 (0.00, 1.79)	0.08 ± 0.01 (0.00, 0.61)	0.07 ± 0.01 (0.00, 0.53)	0.05 ± 0.01 (0.00, 0.68)
Zn	0.01 ± 0.00 (0.00, 0.23)	0.00 ± 0.00 (0.00, 0.01)	0.08 ± 0.00 (0.00, 0.25)	0.00 ± 0.00 (0.00, 0.03)
Zr	0.06 ± 0.03 (0.00, 2.78)	0.02 ± 0.01 (0.00, 0.66)	0.03 ± 0.02 (0.00, 1.49)	0.01 ± 0.01 (0.00, 0.77)

Means ± SE are combined for EDS measurements, regardless of location on the ovipositor (n = 119 measurements for *Magicicada cassinii*, n = 7 individuals; 136 for *M. septendecim*, n = 8; 68 for *M. septendecula*, n = 4; 136 for *Neotibicen linnei*, n = 8). Minimum and maximum measurements are given in parantheses. The four most abundant elements within each species are in bold. Elements are arranged alphabetically.

Elemental concentrations (%wt), with the exception of Cu, Fe, Mn, and Na, were significantly different between the dorsal and ventral surface of the ovipositor of some species (Supplementary Table S2). For those with significantly different inorganic element concentration values between dorsal and ventral surfaces, concentrations were greater on the dorsal surface, except for P and Zn (Supplementary Table S2). Likewise, there were differences in elemental concentrations of the GVIII compared to the GIX on both dorsal and ventral locations. Dorsally, only Cu, K, Zn, and Zr did not show significant differences. When significant differences were present the elements were in greater concentrations on the GVIII, except for Ca, Cl, Mg, Na, P, and S (only for *M. cassinii*) (Supplementary Table S3). On the ventral side only Mg, Mn, and S were individually significant different and were present in greater concentrations on the GIX, except for Mg, which was in greater concentrations on the GVIII (Supplementary Table S4).

Considering all elements across the ovipositor, species differed in total concentrations on the dorsal surface (MANOVA Wilk’s λ = 0.432, F = 5.859, α < 0.001) and the ventral surface (MANOVA Wilk’s λ = 0.506, F = 3.136, α < 0.001). However, there was variation with respect to which specific elements differed between species dorsally and ventrally. On the dorsal surface, significant differences among species were found in eight of fourteen elements (Ca, Cl, Mg, Mn, Na, P, S, Si; Supplementary Table S5), while significant differences on the ventral surface were found in six elements (Cl, K, Mn, Na, P, and Zn; Supplementary Table S6).

**Figure 4:**
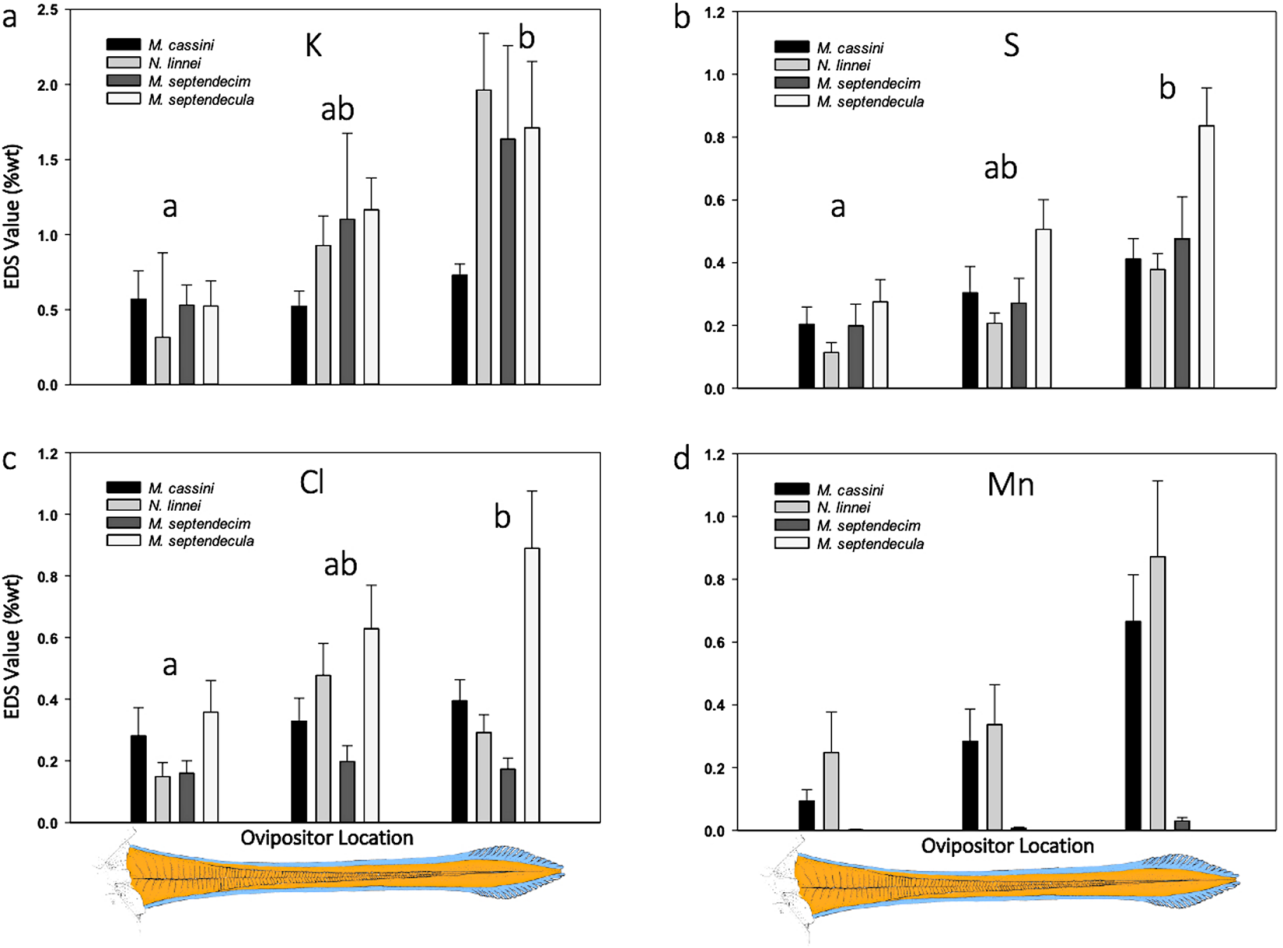
Plot of EDS values averaged across species for the elements K (**a**), S (**b**), Cl (**c**), and Mn (**d**) on the dorsal surface of the ovipositor. Bars ($\overline{X}$ + SE) are plotted in their approximate proximal to distal sampling location along the ovipositor (x-axis). Sampling points 9 and 10 are proximal, sampling points 7 and 8 are mid-ovipositor, and sampling points 1–6 are distal (see Fig. 2 for sampling locations). The EDS values for each species were averaged together per location and tested for significant differences among proximal to distal location groupings at the α = 0.05 level (significant differences are represented by lowercase letters). Mn was not detected in *M. septendecula* on the dorsal surface; therefore, that bar is not shown in graph D nor was Mn included in the tests for significant differences. Note that the y-axis has a different scale for each element.

Element concentration changed along the length of the ovipositor. For four common elements, significantly higher concentrations were found in the distal end of the ovipositor when compared to the proximal region for K, S, and Cl (ANOVA on Ranks for K: H = 9.936, p = 0.006; S: H = 19.856, p < 0.001; Cl: H = 9.445, p = 0.007) and near-significant differences were found for Mn (H = 5.893, p = 0.053). When analyzed by species, the general trend of higher concentrations on the distal end of the ovipositor is apparent, even when differences are not significant (Fig. 4). A similar trend was observed in seven other elements (P, Ca, Si, Mg, Na, Fe, and Zn) with Mg and Na being significantly more abundant in the distal region. The elements Al, Zr, and Cu, although present in the cuticle, did not show this trend.

## Discussion

Due to their distinctive appearance, vociferous songs^31^, and unusual life history traits^32,33^, few animal species are as noteworthy as cicadas, particularly the periodical cicadas (*Magicicada* spp.). The brief surge of periodical cicada adults during mass emergences has a large impact on local ecology. For example, mass emergences create resource pulses, an infrequent event of overabundance among individuals of a species, which can provide enormous resource availability^34^. Periodical cicadas potentially represent the largest biomass of any North American herbivore in deciduous forests^35^; therefore, these resource pulses result in the satiation of predators^36,37^ and provide indirect benefits to plants because of improved soil habitat (e.g., nitrogen availability and microbial biomass)^34^.

Although the mass emergence of periodical cicadas provides the ecological benefits mentioned above, the surge of cicadas also can potentially inflict damage to trees through feeding on xylem by the nymphs from the roots, wood-boring oviposition, and the feeding on xylem by adults^32,38^. Regarding damage via oviposition, female cicadas make linear slits in stems, which, in addition to providing entrance points for pathogens^39^, also cause the phenomenon termed “flagging”, where the portion of the stem that is distal to the oviposition slit rapidly droops, loses its leaves, and dies^32,38^. Although flagging is extensive during mass emergences, the loss of the distal portion of the stems apparently has little effect on plant fitness^38^.

Cicada ovipositors consist of a wide array of inorganic elements in the cuticle, with higher concentrations in the rasping region. Most studies of metal-enriched cuticle report the presence of only a few metals, particularly the transition metals: Fe, Mn, Cu, and Zn^10,16,40^. Here, we report the presence of five transition metals (Cu, Fe, Mn, Zn, and Zr), one post-transition metal (Al), two alkaline Earth metals (Ca and Mg), two alkali metals (K and Na), one metalloid (Si), two non-metals (P and S), and one halogen (Cl) (Table 1). It is unclear if other elements were recorded in the cuticle of previously studied arthropod structures and were not reported, but as far as we know, this is the most diverse array of inorganic elements reported in an insect structure. The only other investigation we are aware of that reported a wide array of elements studied the composition of the intertidal pseudoscorpion (*Halobisium occidentale*), where 11 inorganic elements were reported^41^.

The role of each of these elements in augmenting insect cuticle is not completely understood. Zn is arguably the most well studied element that strengthens cuticle^42,43^ and is reported in the mandibles and ovipositors of insects^8,10,44,45^, the mouthparts of polychaete worms (*Nereis* sp.)^12^, spider fangs^11,46^, and pseudoscorpion structures^41^. Nanoindentation experimentally demonstrated that Zn increases hardness (defined as a material’s resistance to permanent shape changes when a compressive force is applied) of cuticle^47,48^. The reported values for Zn concentrations in structures of other organisms, however, are typically higher than the concentrations measured here. For example, Schofield and Lefevre^11^ found that the tips of some spider fangs have 15% Zn, Fawke *et al*.^15^ found 4.5% and 7.1% Zn localized in the mouthparts of *Atta sexdens* and *Nereis virens*, respectively, and over 20% was found in the mandibles of some Hymenoptera^6^. The average value of Zn recorded here was 0.0003%, with the highest value of only 0.25%, (Table 1), indicating that either only small amounts of Zn are necessary for increased hardness, that Zn does not contribute to hardness in cicada ovipositors, or that supplementing small quantities of Zn with the diversity of elements recorded here provides other benefits to the material properties of cuticle.

Mn, which is sometimes found to co-occur with Zn^6,10,47^, is another transition metal commonly reported in cuticle^15,16,19,45^, but its contribution to the material properties is not fully understood. Broomell *et al*.^49^ reported that Mn increases hardness, and due to its chemical properties, it has the ability to create a diversity of complexes with a large number of protein ligands, but other reports indicate that Mn does not increase hardness^10,16^. In this study, Mn was the most common transition metal recorded with a mean value of 0.22% and a maximum value of 9.51% (raw data not shown), which are similar to the measured values in the ovipositors of some gall-forming Hymenoptera^6^. Also, Mn was in higher concentrations near the rasping region of all studied cicada species, except for *M. septendecula*, where it was absent (Fig. 4). The lack of Mn in ovipositors of *M. septendecula* could be because the individuals studied here lacked Mn in their diet or because they lack the mechanism to sequester and transport it to the ovipositor. Finding Mn localized near particular regions of a structure is not uncommon, as it was observed in the cutting edges of beetle mandibles^16,45^ and termite mandibles^15^, though in generally lower quantities than those observed here. Although the contribution of Mn to cuticular hardness is questionable, it might play another role, such as increasing the resistance to fracture^16,45^. Mn was present in substantial concentrations in three of the four studied cicada species; however, the other commonly reported transition metals (e.g., Fe, Zn, and Cu) were rare or in concentrations lower than previous reports^12,41,49,50^.

Ca is often found in the cuticle of arthropods, particularly crustaceans, where it combines with P as either calcium carbonate, amorphous calcium phosphate (ACP), calcite, and apatite^51^. The use of Ca in the strengthening of cuticle, however, is rare in insects, but is found as amorphous calcium phosphate on the exoskeleton of some larval Diptera^18^. Evidence for the presence of ACP can be inferred from comparing P and Ca ratios, which are approximately 1.5–1.9^52^. Cicada ovipositors did not express a ratio of P to Ca for ACP, except for the mean P and Ca concentrations for the ventral side of *M. cassinii* (Supplementary Table S6), but the presence or absence of this ratio does not necessarily preclude the presence of ACP.

Halogens, such as Cl, are often found in high concentrations along with other elements in arthropod cuticle^4,7,12,16,40^. The high abundance of Cl in this study suggests that Cl plays an important role in augmenting cuticle. Previous studies report Cl to Zn ratios of over 2^12,16^; however, the low values of Zn reported here indicate that Cl, if binding to Zn, also is binding to other elements, perhaps to Mn. Cl to Zn ratios, however, do not have to be at a set ratio and both might be essential to the material properties^16^. The high amounts of Mn and Cl provide evidence for these molecular complexes, but the high values of Cl coupled with the absence of Mn in *M. septendecula* suggests that different elements are bound to Cl. Cl also could be present in other compounds, such as chlorotyrosines, which might co-occur in areas of higher sclerotization on numerous insect species; however, the relationship between chlorotyrosines and the mechanical properties of cuticle requires further exploration^53^.

The contribution of the other reported elements to the material properties is unknown. Si, for instance, has been reported in termite mandibles^54^, but its role has not been determined. The colocalization of inorganic elements, such as Mn and Ca observed here and previously reported^43^, create a range of molecular diversity that would affect the material properties. In addition, the adaptive value of other elements, such as K, which was in high abundance in this study, requires further exploration. Although there are general patterns found in the elemental composition of the cuticle, there are instances where using EDS can give false readings, particularly when the elements are in low concentrations^55^. For example, Zr, which was present in low concentrations here, has a signature peak in EDS analysis that is close to Pt, which was used to coat the specimens in this study. However, given that Zr was present in some species and not in others provides validity for these results.

Although the process of oviposition was not explored here, the combination of previous observations of oviposition with our discoveries here provide insight into how cicada oviposition functions at the proximate level. Cicada ovipositors penetrate through wood by performing antiparallel movements with the GVIII. The antiparallel movements are possible via the fitting of ridges and grooves between the GIX and GVIII (Fig. 3). Antiparallel movements provide a method for penetration, as also observed with the proboscises of fruit-piercing and blood-feeding vampire moths (*Calyptra* spp.)^56^ and the ovipositors of stinging Hymenoptera^57^. The antiparallel movements of the GVIII are likely reinforced and stabilized because the two GVIII connect via interlocking medial ridges (Figs. 1 and 3).

The cicada species studied here have ovipositor structures similar to other cicada species^27^. The *Magicicada* spp., for example, belong to the same subfamily (Cicadettinae) as four of the species studied by Zhong *et al*.^27^ and have a similar structural profile, including the number of teeth, arrangement of medial ridges on the GIX, and the overall appearance. The individuals of *N. linnei* also have a similar number of teeth and profile as other Cicadinae^27^. These findings indicate that some morphological structures, such as teeth number, are useful as a tool for studying cicada phylogeny.

Inorganic elements are localized at various regions along the ovipositor length and differ dorsally and ventrally (Supplementary Table S2) and per structure (Supplementary Tables S3 and S4). The majority of the metals, the halogen Cl, and the non-metal S were significantly more abundant on the dorsal side of the ovipositor compared to the ventral side, where Zn and Si were more abundant; however, this general pattern did show exceptions in some species (Supplementary Table S2). In addition, with some exceptions, inorganic elements were more abundant on the dorsal side of the GVIII compared to the GIX (Supplementary Table S3). The pattern clearly indicates that the majority of the inorganic elements are found on the dorsal side of the ovipositors and that metals are concentrated on and near the teeth of the GVIII in the rasping region. The dorsal side of the teeth is where most of the morphological modifications are present, including the rough surface on the proximal side of each rasping tooth (Fig. 2). We interpret the adaptive value of the smooth distal surface of the teeth as a means to penetrate into the wood, whereas the rough surface of the proximal side of the teeth as a means to grip the wood, similar to the barbs on bee stingers^57^, so that the ovipositor can penetrate deeper into the wood. Interestingly, the Mn and S are in higher concentrations on the ventral side of the GIX, which might assist in enhancing the ability for the GIX to get deeper into the wood.

The ovipositor is not the only structure of the cicada that bores into wood; therefore, other structures might be augmented with inorganic elements. The nymphal and adult stages of the cicada feed on xylem, which would involve the mouthparts penetrating into wood. In addition, given the amount of force necessary for the mouthparts and the ovipositor to push through wood, it could be hypothesized that the tarsi, which grip the wood, also are augmented with inorganic elements. Considering that numerous structures on cicadas are responsible for piercing wood, studying the composition of inorganic components of the cuticle on various parts of the body would be an interesting future direction for research.

Inorganic elements, particularly metals, have been found in the cuticle of numerous arthropod species, and within insects, metal deposition dates back at least 46 million years^58^; however, the mechanism for metal deposition is still unknown^5,59^. Evidence suggests that metals are initially acquired through diet, as indicated in studies with *Rhyzopertha dominica* (Coleoptera) that had their diet supplemented with manganese and zinc^45^. Given the large array of inorganic elements found in cicada ovipositors, cicadas likely sequester the elements from the xylem of plant roots while they feed as nymphs, assuming that metal deposition occurs during cuticle formation and sclerotization^40^. How the elements get stored and subsequently deposited in the cuticle of specific structures requires further study. Previous experiments with ants in metal-polluted regions have found that some elements get sequestered from the environment and stored in the midgut and Malpighian tubules^60^, but how the metals are subsequently transferred to the cuticle of particular structures is not known. One common part of this pathway might be the presence of pores on the structures enhanced with inorganic elements^16,40,43,46^. The differences in inorganic elements among cicada species (Tables S5 and S6) might be related to oviposition and feeding preferences for different plant species; however, this requires further exploration. There also might be a correlation between the material properties of the ovipositors and the plant used for oviposition. A similar relationship was found in the material properties of damselfly ovipositors and oviposition substrates, where damselflies with stiffer ovipositors prefer use plant species with stiffer tissues^61^.

Studies of the material properties of specialized structures, or “tools”^40^, are a good source of bioinspiration and biomimetics. Metal enhanced cuticle, for instance, has lower material density, but hardness values that exceed many human-made polymers^16,43^. Additional studies of the interactions of elements and how they contribute to the material properties of cuticle, through either cross-linkage or other molecular interactions, and the hierarchical nature of cuticle are needed. Material properties, such as hardness, stiffness, and tensile strength are all influenced differently based on the elemental composition^62^, therefore, with additional studies, metal-enhanced structures of insects can inspire new tools that currently lack a human-engineered analog. The anti-parallel movements of cicada ovipositors coupled with the morphology of the rasping region and the augmented cuticle could inspire novel tools for penetrating tissues or other hard surfaces.

## Methods

### Species

Four species of cicadas (Hemiptera: Cicadidae) were used to test for the presence and distribution of inorganic elements in ovipositors. We selected Linne’s annual cicada, *Neotibicen linnei* (Smith and Grossbeck, 1907) (n = 8), and three species of 17-year periodical cicada, the pharaoh cicada, *Magicicada septendecim* (Linnaeus, 1758) (n = 8), the dwarf periodical cicada, *M. cassinii* (Fisher, 1852) (n = 7), and *M. septendecula* Alexander and Moore, 1962 (n = 4). Representative individuals of *N. linnei* were collected in West Lafayette, IN (N40°25′, W86°54′) during September, 2004, *M. septendecim* were collected either in Lake Hope State Park, OH (N39°19′, W82°20′) during March, 1999 or in St. James State Park, NC (N35°45′, W81°53′) during March, 2000, *M. cassinii* were collected in Cincinnati, OH (N39°60′, W84°30′) during June, 2004 or March, 1991, and *M. septendecula* also were collected in Cincinnati, OH in March, 1991. All specimens were collected by hand or with an insect net on or near trees, then pinned and stored in an insect collection kept at Mount Saint Joseph University, Cincinnati, OH.

### Scanning electron microscopy

Cicada ovipositors were removed from the abdomen using dissecting scissors and the lateral sides were imaged with a digital microscope (Dino-Lite Pro AD4113T) at 20X magnification to measure total ovipositor length (Supplementary Fig. S2). The ovipositors were then placed onto an aluminum stub with carbon graphite tape so that the dorsum was visible. Ovipositors were coated in 7 nm of platinum with a sputtercoater (EMS 150R S, Electron Microscopy Sciences) and imaged with scanning electron microscopy (SEM) (JEOL 6010LA) at 15 kV. The ventral sides of the ovipositors also were imaged using the same protocol. Composite images of ovipositors (70X magnification) were assembled in Microsoft PowerPoint and saved as a single image in Adobe Photoshop CS2 (Adobe Systems) (Supplementary Fig. S4). ImageJ software (http://imagej.nih.gov/ij/)^63^ was used for measurements. Selected regions of the dorsal and ventral sides of ovipositors were imaged at higher magnifications.

For consistency, we use the terminology provided by Moulds^26^ and employed by Zhong *et al*.^27^ in their comparative studies of cicada ovipositor structures. Ovipositor length was measured from the base of a GVIII to the distal tip. A total of 10 structural measurements were recorded from the dorsal view of the ovipositor using the composite SEM images. Here, we use the term “rasping region” to refer to the distal region of GVIII where the cuticular projections, referred to hereafter as “teeth” (singular “tooth”), are present and are interspersed by grooves that extend proximally and medially (Fig. 2). Rasping region length was measured from the distal tip of a GVIII to the most proximal groove. The number of teeth on a single GVIII was recorded. The width of a single GVIII and total ovipositor width were measured at four locations (Supplementary Fig. S2). Three width measurements were recorded from the rasping region, which were determined by dividing the rasping region into three equal lengths, then measuring the width at each division and at the base. The ovipositor width also was measured at the middle of the total ovipositor length (Supplementary Fig. S2). Forewing length measurements were recorded as a potential indicator of body size.

### Energy dispersive x-ray spectroscopy

The elemental composition of the cuticle of cicada ovipositors was acquired using energy dispersive x-ray spectroscopy (EDS) (X-Max50, Oxford Instruments). The elemental composition was determined by analyzing a defined region of the ovipositor with EDS for 10 minutes (20 kV, spot size 61, magnifications >200x). The percentage weight for each detected element (determined by Aztec software) was recorded. We analyzed ten dorsal and seven ventral locations along the length of each ovipositor (Supplementary Fig. S2). On the dorsum, six locations were analyzed in the rasping region, including three measurements near the distal tip (one measurement on a GIX, two measurements on a GVIII), and three measurements near the middle (one on a GIX, two on a GVIII). In addition, two measurements at the middle of the total ovipositor length (one on a GIX, one on a GVIII) and two measurements at the base of the ovipositor (one on GIX, one on GVIII) were recorded. On the ventral side of the ovipositor we measured two locations on a GIX in the rasping region, one at the distal tip and the other near the middle. The GVIII was measured at five locations, including the distal tip, the middle of the rasping region, the ventral lobe (=tongue-like slice)^27^ (near where the eggs exit the ovipositor), the middle of the ovipositor length, and the base of the ovipositor (Supplementary Fig. S2).

### Statistics

Analysis of covariance was used to compare species with regard to ovipositor length, adjusting for forewing length as a proxy for body size. Analysis of variance (ANOVA) was used to test for significant differences (p < 0.05) in structural measurements among species. Significant differences in means among species were compared using a Tukey-HSD post hoc test.

A multivariate analysis of variance (MANOVA) was used to test for significant differences (p < 0.05) in chemical composition of the cuticle among species. Analyses were conducted for all elements combined, ovipositor surfaces (dorsal and ventral), and ovipositor locations (GVIII and GIX). Significant differences in means among species were compared using a Tukey-HSD post hoc test. In addition to descriptive statistics for each species and their elemental composition, a Principal Component Analysis (PCA) was used to correlate chemical composition with body size. A PCA was run separately on %wt for each element as well as all morphological variables, with all species combined. A Pearson correlation (p < 0.05) was then run on PCA Axis 1 of the chemical composition of the cuticle vs. PCA Axis 1 of the morphological variables in order to quantify the role of body size with elemental composition. In order to qualify the species specificity, or lack thereof, of cuticular inorganic elements, species were plotted in ordination space as a function of their chemical composition using non-metric multidimensional scaling. Finally, elemental composition was quantified from proximal to distal along the length of the ovipositor. Prior to analysis, sampling locations were grouped as either proximal, middle, or distal and data were combined for each location. On the dorsal surface of the ovipositor, locations were grouped as follows: proximal (9 and 10), middle (7 and 8), and distal (1, 2, 3, 4, 5, 6). On the ventral surface of the ovipositor, locations were grouped as follows: proximal (7), middle (6), and distal (1, 2, 3, 4, 5) (Fig. 2). “Given the resultant differences” in sample sizes after combining location data, the homogeneity of variance for the combined data was violated; therefore, data were compared using a non-parametric Kruskal-Wallis one-way ANOVA on ranks (p < 0.05) with a Dunn’s Method post hoc test.

## Supplementary information

Supplementary information

## Data Availability

The datasets generated during and/or analyzed during the current study are available from the corresponding author on reasonable request.
