# Supplementary material for: An augmented wood-penetrating structure: Cicada ovipositors enhanced with metals and other inorganic elements
Source: Sci Rep. 2019 Dec 24;9:19731. doi: 10.1038/s41598-019-56203-6 (PMC6930284; doi:10.1038/s41598-019-56203-6)
Supplement: Supplementary file 1 — Supplementary information [file 41598_2019_56203_MOESM1_ESM.pdf]

Supplementary Information

An augmented wood-penetrating structure: Cicada ovipositors enhanced with metals and other non-chitinous elements

Matthew S. Lehnert[1], Kristen E. Reiter[1,2], Gregory A. Smith[1], and Gene Kritsky[3]

[1]Department of Biological Sciences, Kent State University at Stark, North Canton, OH 44720 USA

[2]Department of Entomology, University of Illinois at Urbana-Champaign, Urbana, IL 61801 USA

[3]School of Behavioral and Natural Sciences, Mount St. Joseph University, Cincinnati, OH 45233 USA

***Corresponding author:** mlehner1@kent.edu

Supplementary Note

European settlers to North America first noticed the periodical cicadas when the insects emerged in 1633 or 1634 (1). The earliest mention of periodical cicadas' ovipositors in writing was in 1666 when Henry Oldenburg stated that periodical cicadas "had a kind of taile [sic] or sting, which they struck into the tree, and thereby envenomed and killed it" (2). During the 1715 emergence, Reverend Andreas Sandel (3) noted the destruction to trees was the result of egg laying. He wrote, "[cicadas] were also destructive, making slits in the bark of the trees, where they deposited their worms, which withered the branches." Details of the ovipositor were presented in 1734 to the Royal Society in London in a manuscript submitted by Paul Dudley, who had observed the 1699, 1716, and 1733 emergences of the now extinct Brood XI near Boston. Of the ovipositor, Dudley reported, "The female has a small launce in her tail covered and secured with a sheath; this is what she makes use of to open a place in the twigs or branches of trees; where she lays her eggs. This launce is about half an inch long, small as the finest pin and shaped like a spear" (1). Linnaeus' student, Pehr Kalm (4), observed the 1749 emergence, which permitted him to note for the first time that the ovipositor was made of two "darts" and that it was thick and sharper at the distal end.

Details of the ovipositor were published by Nathanial Potter (5) (Supplementary Fig. S1), although most of this account was likely written by Gideon B. Smith. The section on the female ovipositor reads:

> The most remarkable parts of this extraordinary insect are those of the female - the machinery by which she works in effecting a depository for her eggs. The *ovipositor* is lodged in a groove, and lies at *the terminating ring of the abdomen*, and the *oviduct* passes

> through its *centre*. When not in use, it is concealed, except a minute point at its lower extremity. It is composed of *three distinct pieces*, connected at the sides *by the nicest tongue and groove*, by which *the two side pieces play up and down upon the centre piece. The edges and points of the side pieces are serrated, forming perfect saws*, while their *flattened surface* form *rasps* equally perfect. When the instrument is protruded and examined by the microscope, the denticulations of the saws are clearly perceived. There are *fifteen teeth* on each side, symmetrically arranged, tapering away in fineness towards the point, where we find *three or four* more, so small that it is scarcely possible to determine, by the best glasses, whether there are *three* or *four*. In some there appeared to be three, in others four.

This description is remarkably accurate, describing the three parts of the "ovipositor" and the tongue and groove articulation that allows the two "saws" to stay in alignment and move alternatively with respect to each other.

Supplementary Figures

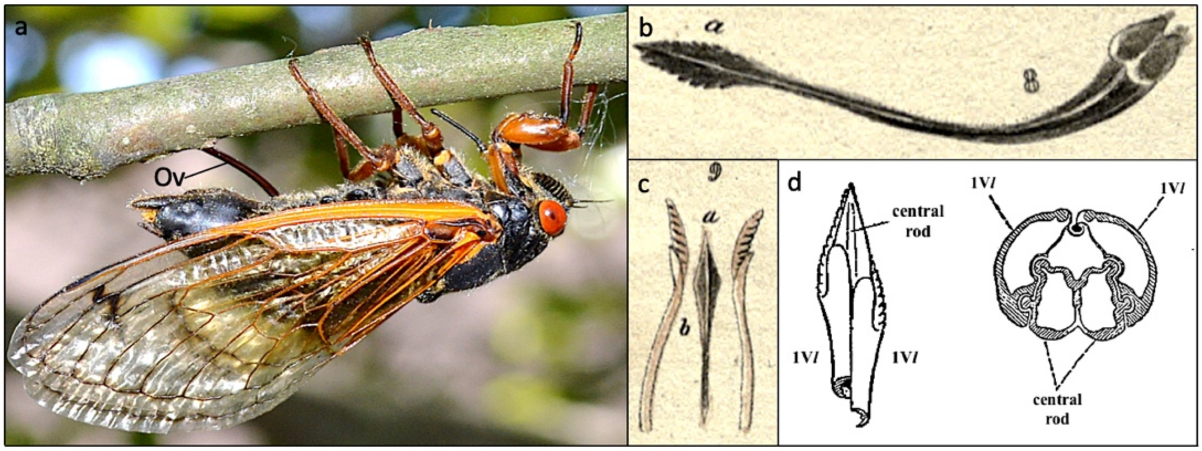

Supplementary Figure S1. Cicada oviposition and early illustrations of the ovipositor. (a) A female periodical cicada, *M. cassinii*, using its ovipositor (ov) to penetrate through wood. (b, c) Two hand-drawn illustrations of the ovipositor in Potter's pamphlet, which show the thick serrated saw at the distal end. The second illustration (c) as described by Potter (5) shows the "parts of the ovipositor separated … showing the form of the saws and rasps (*a*), and of the oviduct." (d) Illustrations of the ovipositor by Snodgrass (6). The left image in (d) shows a ventral view of the antiparallel movements of the first valvulae (1V*l*) (=gonapophyses VIII, 7) along the central rod (=gonapophyses IX). The illustration of the cross section, shown on the right, details the fitting mechanism of the central rod with the 1V*l*.

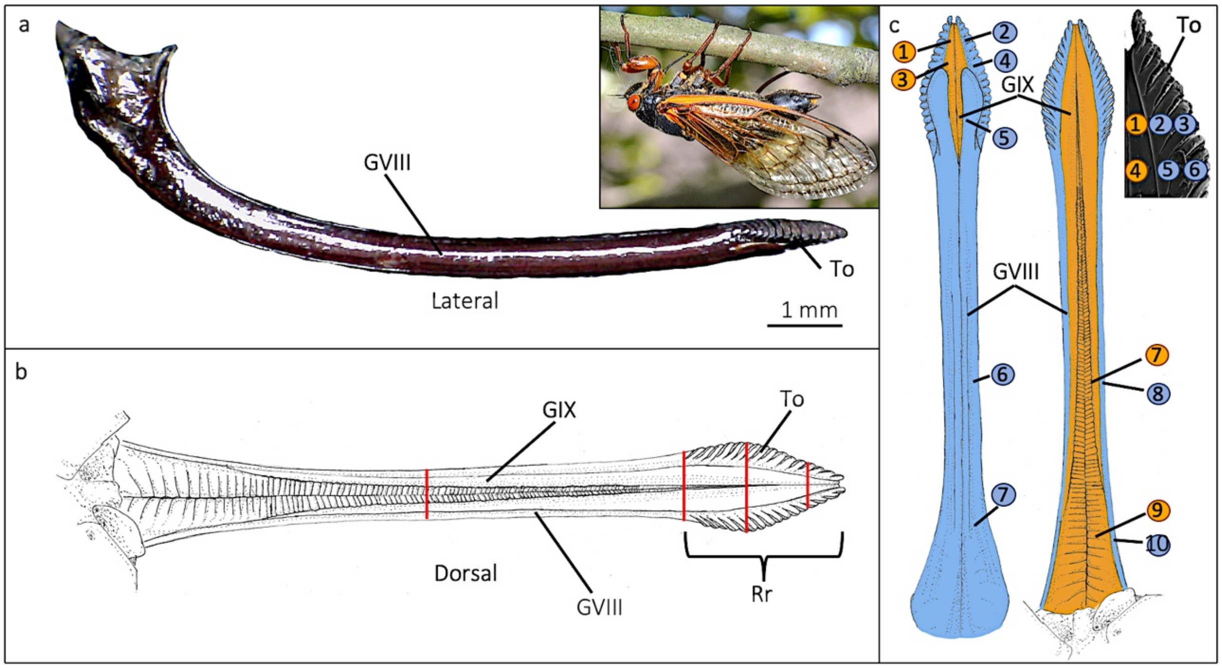

Supplementary Figure S2. Images of the cicada ovipositor showing locations of measurements. (a) Image of the lateral view of the ovipositor acquired with a digital microscope showing the gonapophyses VIII (GVIII) and the distal tip with teeth (singular= tooth, To). (b) Illustration of the dorsal view of *M. cassinii* ovipositor showing GVIII and gonapophyses IX (GIX), and the rasping region (Rr). The red lines indicate where the ovipositor widths were measured. (c) Illustrations of the ventral (shown on left) and dorsal (right) sides of the ovipositor of *M. cassinii*. The GVIII and GIX are shown in blue and orange, respectively. The numbered circles correspond to the locations along the ovipositor where EDS measurements (%wt) were acquired. The ovipositors were illustrated by Brooke Pandrea.

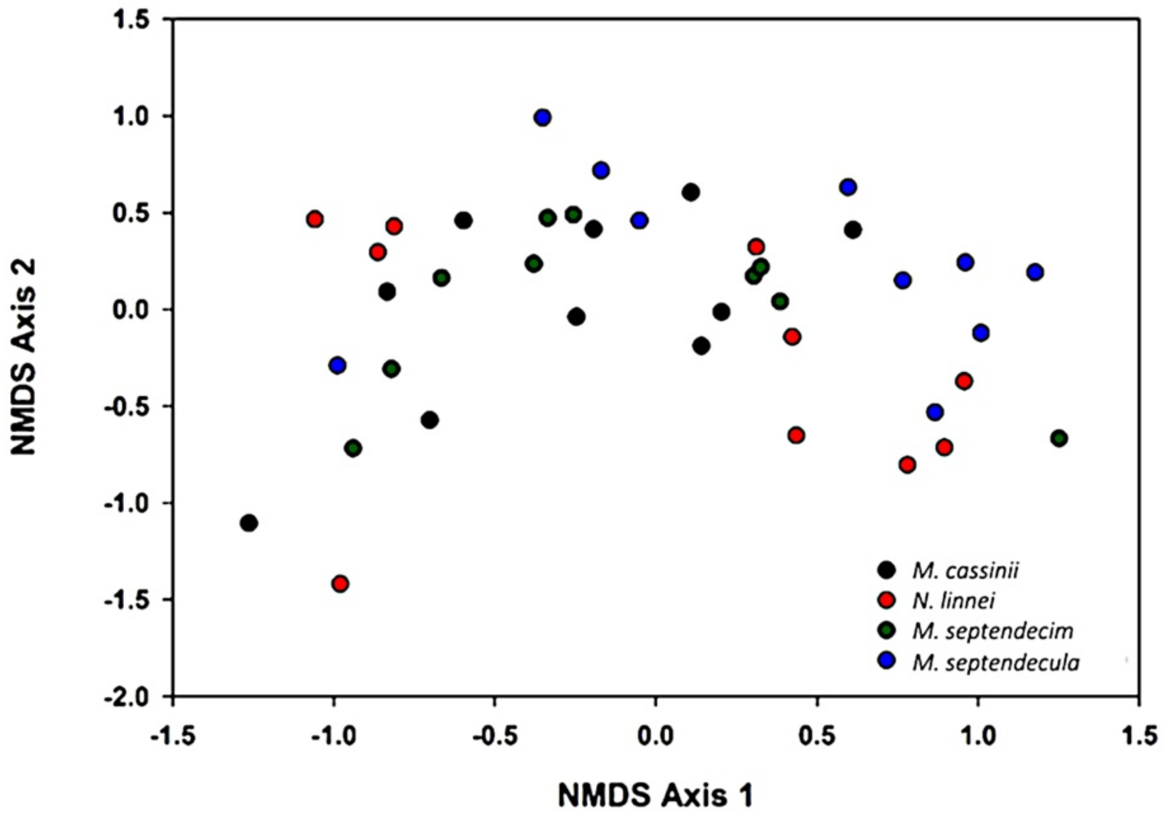

Supplementary Figure S3. Plot of individuals of four cicada species within ordination space. Individuals are located on the plot as a function of elemental composition of the ovipositor using Non-metric Multidimensional Scaling. Species are color-coded as per the legend.

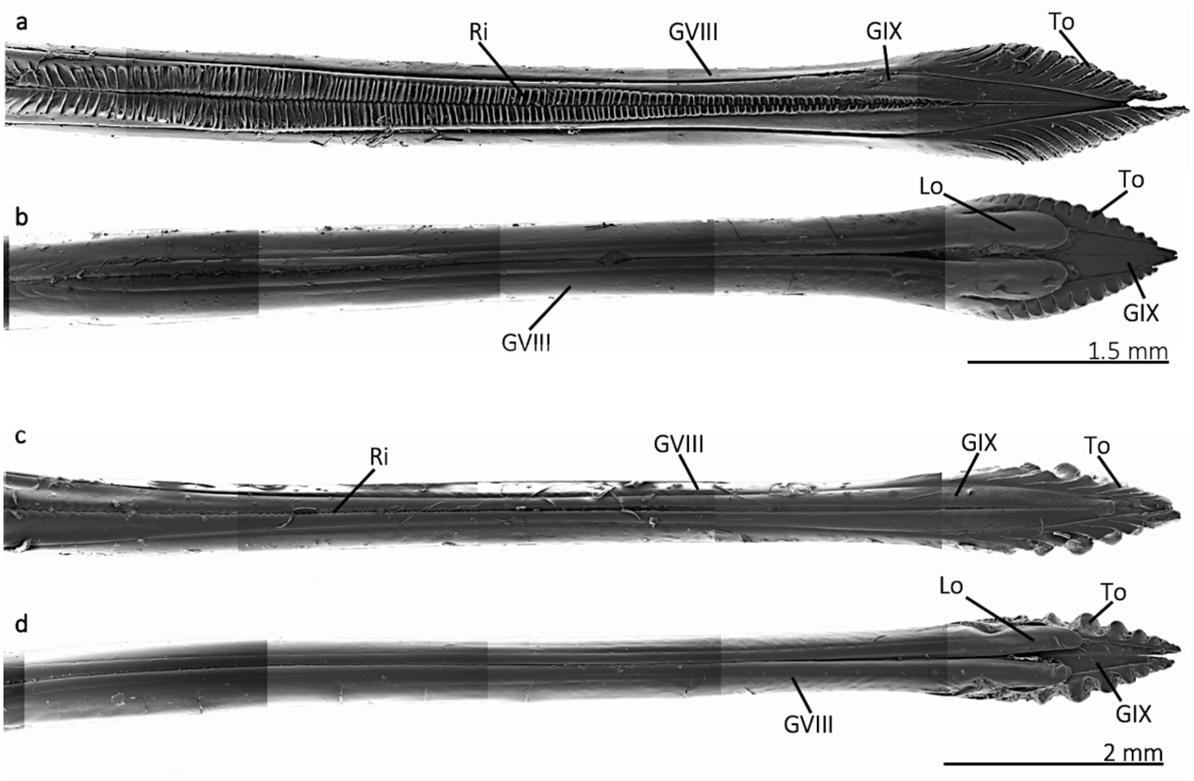

Supplementary Figure S4. SEM composite images of cicada ovipositors. All images show approximately 80% of the distal region of the ovipositor. Composite images were assembled from serial images of ovipositors acquired at 70X magnification. (a) dorsal view of an ovipositor of Magicicada septendecula showing the lateral gonapophyses VIII (GVIII) with the teeth (tooth = To) at the rasping region. The medial gonapophyses IX (GIX) have interlocking ridges (Ri). (b) The ventral side of an ovipositor of M. septendecim shows the lobes of the GVIII near the distal region. The dorsal (c) and ventral (d) sides of *Neotibican linnei* ovipositors.

Supplementary Tables

Supplementary Table S1. Comparison of ovipositor measurements (μm, Mean±SE) among cicada species. Letters represent significant differences ($p<0.05$) for each measurement among species using a Tukey HSD post hoc.

Species	n	FWL	Ovipositor length	Ovipositor width at midpoint*	Rasping region length	Rasping region width at base*	Rasping region width (proximal)*	Rasping region width (distal)*	teeth #
Magicicada cassinii	7	30.00 ±0.22c	11211.29 ±254.65b	477.73±11.30 (138.34±14.44)	1565.19± 41.39c	643.78±8.04b (168.63±6.78b)	807.79±18.37ab (272.09±12.49)a	566.85±16.29ab (204.95±6.60)	15.86 ±0.14a
M. septendecula	4	30.00 ±0.82c	10988.50 ±470.00b	445.77±39.92 (130.38±21.99)	1502.60± 46.89c	641.92±34.14b (166.50±10.27b)	740.28±23.88ab (281.08±14.79)a	490.32±28.06b (195.00±13.51)	15.25 ±0.25a
M. septendecim	8	35.63 ±0.42b	13351.00 ±216.72a	533.07±28.40 (189.25±14.89)	1795.02± 38.93b	739.58±29.25a (229.46±21.90a)	862.61±57.57a (319.99±26.31)a	683.88±57.82a (237.54±15.52)	16.00 ±0.27a
Neotibicen linnei	8	41.25 ±0.31a	12835.75 ±196.29a	449.44±16.15 (142.62±9.83)	2079.30± 46.68a	460.85±12.81c (95.37±6.47c)	644.63±19.33b (167.90±6.97)b	677.71±30.44a (222.91±8.26)	11.00 ±0.19b

*Width of only GVIII shown in parentheses

Supplementary Table S2. Element composition (%wt) within the cuticle of the ovipositors of four cicada species. Means ± SE are combined for EDS measurements on either the dorsal (n = 70 measurements for *Magicicada cassinii*, n = 7 individuals; 80 for *M. septendecim*, n = 8; 40 for *M. septendecula*, n = 4; 80 for *Neotibicen linnei*, n = 8) or ventral (n = 49 measurements for *M. cassinii*; 56 for *M. septendecim*; 28 for *M. septendecula*; 56 for *N. linnei*) surface and compared with an ANOVA on ranks. Values in bold indicate significant differences within species between dorsal and ventral element composition at the α = 0.05 level. Elements are arranged alphabetically.

	M. cassinii		*M. septendecim*		*M. septendecula*		*N. linnei*	
Element	Dorsal	Ventral	Dorsal	Ventral	Dorsal	Ventral	Dorsal	Ventral
Al	**0.12 ± 0.03**	**0.02 ± 0.01**	**0.34 ± 0.13**	**0.19 ± 0.07**	**0.02 ± 0.01**	**0.04 ± 0.01**	**0.31 ± 0.10**	**0.01 ± 0.01**
Ca	**0.20 ± 0.04**	**0.07 ± 0.02**	0.05 ± 0.01	0.19 ± 0.03	0.10 ± 0.02	0.06 ± 0.01	0.03 ± 0.01	0.04 ± 0.02
Cl	**0.36 ± 0.05**	**0.14 ± 0.02**	0.18 ± 0.03	0.30 ± 0.04	**0.35 ± 0.09**	**0.14 ± 0.01**	0.73 ± 0.12	0.24 ± 0.05
Cu	0.00 ± 0.00	0.00 ± 0.00	0.00 ± 0.00	0.00 ± 0.00	0.00 ± 0.00	0.00 ± 0.00	0.00 ± 0.00	0.00 ± 0.00
Fe	0.02 ± 0.01	0.01 ± 0.01	0.01 ± 0.00	0.03 ± 0.01	0.01 ± 0.00	0.00 ± 0.00	0.05 ± 0.04	0.00 ± 0.00
K	**0.66 ± 0.08**	**0.25 ± 0.05**	1.31 ± 0.39	1.43 ± 0.24	**0.84 ± 0.16**	**0.40 ± 0.07**	1.36 ± 0.28	0.58 ± 0.28
Mg	0.03 ± 0.00	0.05 ± 0.01	**0.05 ± 0.00**	**0.06 ± 0.01**	0.07 ± 0.01	0.07 ± 0.01	0.08 ± 0.02	0.06 ± 0.01
Mn	0.47 ± 0.10	0.08 ± 0.02	0.02 ± 0.01	0.64 ± 0.15	0.17 ± 0.04	0.02 ± 0.01	0.00 ± 0.00	0.00 ± 0.00
Na	0.01 ± 0.00	0.01 ± 0.00	0.01 ± 0.00	0.04 ± 0.01	0.05 ± 0.01	0.01 ± 0.00	0.01 ± 0.00	0.00 ± 0.00
P	0.12 ± 0.03	0.10 ± 0.04	0.21 ± 0.04	0.02 ± 0.01	**0.03 ± 0.01**	**0.16 ± 0.03**	0.27 ± 0.06	0.11 ± 0.04
S	**0.35 ± 0.04**	**0.20 ± 0.03**	0.38 ± 0.08	0.29 ± 0.03	**0.27 ± 0.02**	**0.23 ± 0.03**	0.66 ± 0.08	0.35 ± 0.09
Si	**0.04 ± 0.01**	**0.08 ± 0.04**	**0.06 ± 0.01**	**0.03 ± 0.01**	0.07 ± 0.02	0.11 ± 0.02	**0.08 ± 0.02**	**0.05 ± 0.02**
Zn	**0.00 ± 0.00**	**0.01 ± 0.00**	**0.00 ± 0.00**	**0.00 ± 0.00**	**0.00 ± 0.00**	**0.00 ± 0.00**	0.00 ± 0.00	0.02 ± 0.01
Zr	0.08 ± 0.05	0.02 ± 0.02	**0.03 ± 0.01**	**0.01 ± 0.01**	0.01 ± 0.01	0.00 ± 0.00	0.04 ± 0.04	0.03 ± 0.02

Supplementary Table S3. Element composition (%wt) within the cuticle on the dorsal side of the ovipositors of four cicada species. Means ± SE are combined for EDS measurements on either the GIX (n = 28 measurements for *Magicicada cassinii*, n = 7 individuals; 32 for *M. septendecim*, n = 8; 16 for *M. septendecula*, n = 4; 32 for *Neotibicen linnei*, n = 8) or GVIII (n = 42 measurements for *M. cassinii*; 48 for *M. septendecim*; 24 for *M. septendecula*; 48 for *N. linnei*) of the dorsal surface and compared with an ANOVA on ranks. Values in bold indicate significant differences within species between the GVIII and GIX element composition at the α = 0.05 level. Elements are arranged alphabetically.

	M. cassinii		*M. septendecim*		*M. septendecula*		*N. linnei*	
Element	GIX	GVIII	GIX	GVIII	GIX	GVIII	GIX	GVIII
Al	0.09 ± 0.04	0.13 ± 0.05	0.15 ± 0.06	0.47 ± 0.21	**0.06 ±0.04**	**0.47 ± 0.16**	0.03 ± 0.01	0.29 ± 0.11
Ca	0.10 ± 0.03	0.26 ± 0.05	**0.07 ± 0.02**	**0.03 ± 0.01**	0.01 ± 0.01	0.04 ± 0.01	0.09 ± 0.03	0.25 ± 0.05
Cl	**0.47 ± 0.08**	**0.28 ± 0.05**	**0.22 ± 0.04**	**0.15 ± 0.03**	0.62 ± 0.25	0.80 ± 0.11	0.23 ± 0.03	0.34 ± 0.07
Cu	0.00 ± 0.00	0.00 ± 0.00	0.00 ± 0.00	0.00 ± 0.00	0.00 ± 0.00	0.00 ± 0.00	0.00 ± 0.00	0.01 ± 0.00
Fe	0.00 ±0.00	0.03 ± 0.01	0.01 ± 0.01	0.01 ± 0.01	0.00 ± 0.00	0.09 ± 0.06	**0.00 ± 0.00**	**0.05 ± 0.02**
K	0.79 ± 0.15	0.57 ± 0.07	1.09 ± 0.34	1.45 ± 0.62	0.95 ± 0.32	1.64 ± 0.41	0.68 ± 0.14	1.92 ± 0.38
Mg	**0.05 ± 0.01**	**0.02 ± 0.00**	0.05 ± 0.01	0.04 ± 0.01	0.05 ± 0.02	0.10 ± 0.03	**0.06 ± 0.01**	**0.06 ± 0.01**
Mn	0.17 ± 0.04	0.68 ± 0.15	0.03 ± 0.02	0.01 ±0.00	0.00 ± 0.00	0.00 ± 0.00	**0.10 ± 0.02**	**1.00 ± 0.24**
Na	**0.02 ± 0.01**	**0.01 ± 0.00**	0.01 ± 0.01	0.01 ± 0.00	0.01 ± 0.01	0.01 ± 0.00	0.06 ± 0.02	0.04 ± 0.01
P	0.18 ± 0.06	0.08 ± 0.02	**0.24 ± 0.05**	**0.18 ± 0.05**	0.19 ± 0.05	0.32 ± 0.09	0.01 ± 0.01	0.04 ± 0.02
S	**0.49 ± 0.08**	**0.25 ± 0.04**	**0.37 ± 0.07**	**0.38 ± 0.13**	0.59 ± 0.16	0.70 ± 0.09	0.23 ± 0.02	0.33 ± 0.06
Si	0.05 ± 0.02	0.03 ± 0.01	0.07 ± 0.02	0.05 ± 0.01	**0.06 ± 0.03**	**0.09 ± 0.01**	0.02 ± 0.01	0.04 ± 0.01
Zn	0.01 ± 0.04	0.00 ± 0.00	0.00 ± 0.00	0.00 ± 0.00	0.00 ± 0.00	0.00 ± 0.00	0.00 ± 0.00	0.00 ± 0.00
Zr	0.14 ± 0.11	0.04 ± 0.04	0.03 ± 0.02	0.02 ± 0.01	0.09 ± 0.09	0.00 ± 0.00	0.02 ± 0.02	0.00 ± 0.00

Supplementary Table S4. Element composition (%wt) within the cuticle on the ventral side of the ovipositors of four cicada species. Means ± SE are combined for EDS measurements on either the GIX (n = 14 measurements for *Magicicada cassinii*, n = 7 individuals; 16 for *M. septendecim*, n = 8; 8 for *M. septendecula*, n = 4; 16 for *Neotibicen linnei*, n = 8) or GVIII (n = 35 measurements for *Magicicada cassinii*; 40 for *M. septendecim*; 20 for *M. septendecula*; 40 for *N. linnei*) of the ventral surface and compared with an ANOVA on ranks. Values in bold indicate significant differences within species between the GVIII and GIX element composition at the α = 0.05 level. Elements are arranged alphabetically.

	M. cassinii		*M. septendecim*		*M. septendecula*		*N. linnei*	
Element	GIX	GVIII	GIX	GVIII	GIX	GVIII	GIX	GVIII
Al	0.00 ± 0.00	0.03 ± 0.02	0.05 ± 0.03	0.04 ± 0.01	0.00 ± 0.00	0.01 ± 0.01	0.03 ± 0.03	0.02 ± 0.01
Ca	0.06 ± 0.02	0.07 ± 0.03	0.10 ± 0.05	0.04 ± 0.01	0.01 ± 0.00	0.05 ± 0.02	0.13 ± 0.04	0.09 ± 0.02
Cl	0.14 ± 0.04	0.14 ± 0.02	0.13 ± 0.03	0.14 ± 0.02	0.32 ± 0.17	0.21 ± 0.02	0.28 ± 0.06	0.38 ± 0.12
Cu	0.00 ± 0.00	0.00 ± 0.00	0.00 ± 0.00	0.00 ± 0.00	0.00 ± 0.00	0.00 ± 0.00	0.00 ± 0.00	0.00 ± 0.00
Fe	0.00 ± 0.00	0.01 ± 0.01	0.01 ± 0.00	0.03 ± 0.00	0.00 ± 0.00	0.00 ± 0.00	0.00 ± 0.00	0.01 ± 0.01
K	0.23 ± 0.05	0.26 ± 0.06	0.34 ± 0.09	0.42 ± 0.09	1.25 ± 0.95	0.31 ± 0.09	0.98 ± 0.28	0.79 ± 0.20
Mg	0.05 ± 0.01	0.05 ± 0.02	0.08 ± 0.02	0.07 ± 0.01	**0.02 ± 0.01**	**0.08 ± 0.02**	0.09 ± 0.02	0.06 ± 0.01
Mn	**0.16 ± 0.08**	**0.05 ± 0.01**	0.03 ± 0.01	0.02 ± 0.01	0.00 ± 0.00	0.00 ± 0.00	**0.35 ± 0.12**	**0.10 ± 0.02**
Na	0.01 ± 0.06	0.01 ± 0.00	0.02 ± 0.01	0.01 ± 0.00	0.00 ± 0.00	0.01 ± 0.00	0.03 ± 0.02	0.05 ± 0.02
P	0.09 ± 0.04	0.10 ± 0.06	0.19 ± 0.05	0.14 ± 0.03	0.16 ± 0.10	0.09 ± 0.03	0.03 ± 0.02	0.04 ± 0.02
S	**0.26 ± 0.05**	**0.18 ± 0.03**	**0.30 ± 0.05**	**0.20 ± 0.03**	**0.63 ± 0.30**	**0.24 ± 0.05**	**0.36 ± 0.04**	**0.23 ± 0.03**
Si	0.16 ± 0.13	0.05 ± 0.01	0.12 ± 0.05	0.10 ± 0.02	0.04 ± 0.02	0.06 ± 0.02	0.09 ± 0.04	0.06 ± 0.02
Zn	0.02 ± 0.01	0.01 ± 0.00	0.01 ± 0.00	0.00 ± 0.00	0.04 ± 0.03	0.01 ± 0.00	0.00 ± 0.00	0.00 ± 0.00
Zr	0.00 ± 0.00	0.02 ± 0.02	0.00 ± 0.00	0.00 ± 0.00	0.00 ± 0.00	0.04 ± 0.03	0.05 ± 0.05	0.00 ± 0.00

Supplementary Table S5. Element composition within the dorsal surface of the cuticle of the ovipositors of four cicada species (*Neotibican linnei*, n = 8; *Magicicada septendecim*, n = 8; *M. cassinii*, n = 7; *M. septendecula*, n = 4). Values are mean %wt ± SE. Letters indicate significant differences between species for metal concentration at the α = 0.05 level with an ANOVA on ranks and Dunn's post hoc test for pair-wise comparisons. Elements are arranged alphabetically.

Element	*M. cassinii* (n = 70)	*M. septendecim* (n = 80)	*M. septendecula* (n = 40)	*N. linnei* (n = 80)
Al	0.11 ± 0.03 ab	0.34 ± 0.13 a	0.31 ± 0.10 ab	0.19 ± 0.07 b
Ca	0.12 ± 0.03	0.05 ± 0.01	0.03 ± 0.01	0.19 ± 0.03
Cl	0.36 ± 0.05 a	0.18 ± 0.02 b	0.73 ± 0.12 c	0.30 ± 0.04 a
Cu	0.00 ± 0.00	0.00 ± 0.00	0.00 ± 0.00	0.00 ± 0.00
Fe	0.02 ± 0.01	0.01 ± 0.00	0.06 ± 0.04	0.03 ± 0.01
K	0.66 ± 0.08 ab	1.31 ± 0.39 a	1.36 ± 0.28 b	1.43 ± 0.24 b
Mg	0.03 ± 0.00 a	0.05 ± 0.00 ab	0.08 ± 0.02 b	0.06 ± 0.01 b
Mn	0.47 ± 0.10 a	0.02 ± 0.01 b	0.00 ± 0.00 b	0.64 ± 0.15 a
Na	0.01 ± 0.00 ab	0.01 ± 0.00 a	0.01 ± 0.00 a	0.05 ± 0.01 b
P	0.12 ± 0.03 a	0.21 ± 0.04 ab	0.27 ± 0.06 b	0.02 ± 0.12 c
S	0.35 ± 0.04 a	0.38 ± 0.08 a	0.66 ± 0.08 b	0.03 ± 0.03 a
Si	0.04 ± 0.01 ac	0.06 ± 0.01 b	0.08 ± 0.02 ab	0.03 ± 0.01 c
Zn	0.00 ± 0.00	0.00 ± 0.00	0.00 ± 0.00	0.00 ± 0.00
Zr	0.08 ± 0.05	0.03 ± 0.01	0.04 ± 0.04	0.01 ± 0.01

Supplementary Table S6. Element composition within the ventral surface of the cuticle of the ovipositors of four cicada species (*Neotibican linnei*, n = 8; *Magicicada septendecim*, n = 8; *M. cassinii*, n = 7; *M. septendecula*, n = 4). Values are mean %wt ± SE. Letters indicate significant differences between species for metal concentration at the α = 0.05 level with an ANOVA on ranks and Dunn's post hoc test for pair-wise comparisons. Elements are arranged alphabetically.

Element	*M. cassinii* (N = 49)	*M. septendecim* (N = 56)	*M. septendecula* (N = 28)	*N. linnei* (N = 56)
Al	0.02 ± 0.01	0.04 ± 0.01	0.00 ± 0.00	0.02 ± 0.01
Ca	0.07 ± 0.02	0.06 ± 0.01	0.03 ± 0.02 a	0.10 ± 0.02
Cl	0.14 ± 0.02 a	0.14 ± 0.01 a	0.24 ± 0.05 b	0.35 ± 0.09 b
Cu	0.00 ± 0.00	0.00 ± 0.00	0.00 ± 0.00	0.00 ± 0.00
Fe	0.01 ± 0.01	0.00 ± 0.00	0.00 ± 0.00	0.01 ± 0.00
K	0.25 ± 0.05	0.40 ± 0.07	0.58 ± 0.28	0.84 ± 0.16 a
Mg	0.05 ± 0.01	0.07 ± 0.01	0.06 ± 0.01	0.07 ± 0.01
Mn	0.08 ± 0.02 a	0.02 ± 0.01 b	0.00 ± 0.00 b	0.02 ± 0.04 a
Na	0.01 ± 0.00	0.01 ± 0.00	0.00 ± 0.00	0.05 ± 0.01
P	0.10 ± 0.04 ab	0.16 ± 0.03 a	0.11 ± 0.03 ab	0.03 ± 0.01 b
S	0.20 ± 0.03 a	0.23 ± 0.03 ab	0.35 ± 0.09 ab	0.03 ± 0.02 b
Si	0.08 ± 0.04 ab	0.11 ± 0.02 a	0.05 ± 0.02 ab	0.07 ± 0.02 b
Zn	0.01 ± 0.00 a	0.00 ± 0.00 b	0.02 ± 0.01 a	0.00 ± 0.00 b
Zr	0.02 ± 0.02	0.00 ± 0.00	0.03 ± 0.02	0.01 ± 0.01

Supplementary References

1. Kritsky, G. Periodical cicadas: the plague and the puzzle. Indianapolis: Indiana Academy of Science (2004).
2. Oldenburg, H. Some observations of swarms of strange insects and the mischiefs done by them. *Philos Trans R Soc Lond*. **8**, 137 (1666).
3. Sandel, A. Extracts from the Journal of. *Pa Mag Hist Biogr.* **30**, 448-449 (1906).
4. Kalm, P. Beskrifning på et slags gräs-hoppor uti Norra America (Cicada *septendecim*). *Vetenskapsakademien*. **17**, 101-116 (1756).
5. Potter, N. Notes on the *Locusta septentrionalis americanae decim septima*. In: J. Robinson J, editor. Baltimore, Maryland. p. 29 (1839).
6. Snodgrass, R. E. Principles of Insect Morphology. New York and London: McGraw-Hill Book Company, Inc. (1935).
7. Moulds, M. S. An appraisal of the higher classification of cicadas (Hemiptera: Cicadoidea) with special reference to the Australian fauna. *Rec Aust Mus.* **57,** 375-446 (2005).
